# CYP2D6 Phenotype as a Predictor of Adverse Drug Reactions in Patients Treated With Trazodone: An Explorative Pharmacogenetic Study

**DOI:** 10.1097/JCP.0000000000002123

**Published:** 2026-01-07

**Authors:** Florine M. Wiss, Christian D. Krieg, Markus L. Lampert, Henriette E. Meyer zu Schwabedissen, Céline K. Stäuble, Thorsten Mikoteit, Christian Imboden, Samuel S. Allemann

**Affiliations:** 1 Pharmaceutical Care, Department of Pharmaceutical Sciences, University of Basel, Basel; 2 Institute of Hospital Pharmacy, Solothurner Spitäler, Olten; 3 Biopharmacy, Department of Pharmaceutical Sciences, University of Basel, Basel; 4 Psychiatric Services Solothurn, Solothurner Spitäler, Solothurn; 5 Department of Medicine, University of Basel, Basel; 6 University Hospital of Psychiatry and Psychotherapy, University of Bern, Bern, Switzerland

**Keywords:** trazodone, mCPP, pharmacogenetic, adverse drug reaction, CYP2D6

## Abstract

**Purpose:**

Trazodone is metabolized by CYP3A4 to the active metabolite meta-chlorophenylpiperazine (mCPP), which is associated with various adverse drug reactions (ADRs). mCPP is further inactivated by CYP2D6. Despite the knowledge of trazodone metabolism, the evidence regarding the impact of pharmacogenetics remains limited. This study aimed to examine the association between pharmacogenetic variants in genes involved in the metabolism of trazodone and the occurrence of ADRs during trazodone treatment.

**Methods:**

We performed an explorative observational study using available data and serum samples from 2 ongoing pharmacogenetic studies. For patients treated with trazodone, we analyzed information on trazodone tolerability, genetic variants in *CYP2D6, CYP3A5,* and *ABCB1*, as well as co-medications to assess potential drug-drug-gene interactions (phenoconversion). Serum levels of mCPP and trazodone were measured in a subset of patients.

**Results:**

Data from 98 patients were analyzed. Reduced CYP2D6 activity was associated with an increased risk of ADRs during trazodone therapy. After accounting for phenoconversion and adjusting for sex, trazodone dose, and CYP3A5 phenotype, CYP2D6 poor metabolizers were found to be more likely to develop ADRs compared with normal metabolizers (OR: 8.96; 95% CI=1.67-48.08). No association with ADRs was found for genetic variants in *CYP3A5* and *ABCB1*. Subgroup analysis revealed that reduced CYP2D6 activity was associated with a higher mCPP-to-trazodone ratio and a greater tendency for ADRs.

**Conclusions:**

Our findings suggest that mCPP could contribute to trazodone-related ADRs, especially in individuals with reduced CYP2D6 metabolism. Larger clinical studies are needed to confirm that *CYP2D6* genotyping could contribute to preventing ADRs in clinical practice.

Pharmacogenetic analyses are applied in personalized medicine to optimize pharmacotherapy for individual patients.^1^ There is growing evidence that consideration of pharmacogenetic information in personalized antidepressant therapy improves effectiveness and tolerability.^2–4^ Several antidepressants are well-studied regarding the influence of pharmacogenetic variants, and pharmacogenetic guidelines exist, such as those provided by the Clinical Pharmacogenetics Implementation Consortium or the Dutch Pharmacogenetics Working Group.^5–8^ One example is escitalopram, a selective serotonin reuptake inhibitor, for which pharmacogenetic guidelines recommend dosing adjustments or switching to another antidepressant depending on the genotype of *Cytochrome P450 2C19* (*CYP2C19*).^5,7^

Another antidepressant for which the evidence regarding the impact of pharmacogenetics remains limited is trazodone. Accordingly, for trazodone no recommendations in pharmacogenetic guidelines exist.

Trazodone belongs to the class of serotonin (5-HT) receptor antagonists and reuptake inhibitors. Specifically, at low doses, it antagonizes 5-HT2A receptors, α-adrenoceptors, and histamine H1 receptors. At higher doses, it also acts as an antagonist of 5-HT2C receptors, and an inhibitor of 5-HT reuptake.^9^ Because of its antidepressant and anxiolytic properties, it is used in treating depression with or without accompanying anxiety disorders.^10,11^ Its sedative effects make low-dose trazodone (25 to 100 mg at bedtime) a common “off-label” treatment for insomnia. It can be used as monotherapy or in combination with other antidepressants. Trazodone is considered an effective and relatively safe antidepressant, especially when compared with tricyclic antidepressants, which are more frequently associated with adverse drug reactions (ADRs) due to their anticholinergic effects.^12,13^

Trazodone is extensively metabolized, mainly involving CYP3A4 enzymes. In this reaction, the active metabolite m-chlorophenylpiperazine (mCPP) is formed (Figure 1).^14^ mCPP has complex signaling effects on various 5-HT receptor subtypes, with a prominent 5-HT2C agonism.^16^ Moreover, there are data, showing that intravenously or orally administered mCPP can lead to a dose-dependent increase in adrenocorticotropic hormone (ACTH), cortisol, and prolactin levels. These hormonal changes are accompanied by increased body temperature, blood pressure, and pulse rate. Nausea, dizziness, tremor, and many other ADRs have also been reported for mCPP.^17–19^ mCPP itself is inactivated by hydroxylation mainly catalyzed by CYP2D6.^15^

**FIGURE 1:**
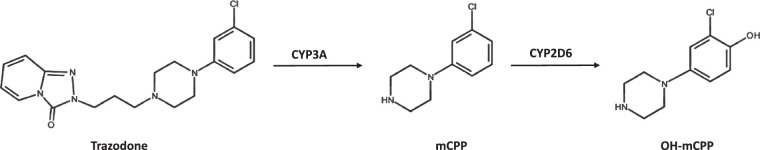
Hepatic metabolism of trazodone and mCPP.^14,15^

The involvement of CYP3A4 and CYP2D6 in the metabolism of trazodone is further supported by findings showing that the formation of mCPP in liver microsomes is significantly reduced in the presence of the known CYP3A4 inhibitor ketoconazole.^20^ In contrast, when trazodone was combined with fluoxetine, a potent CYP2D6 inhibitor, plasma levels of mCPP significantly increased in patients with major depression.^21^ Changes in the activity of the enzymes involved in the formation or metabolism of mCPP due to drug-drug interactions can thus influence the systemically available amount of mCPP. This suggests that genetically determined variations in the activity of CYP3A or CYP2D6 could also affect mCPP serum levels and, consequently, the tolerability of trazodone.

The CYP3A sub-family includes the enzymes CYP3A4 and CYP3A5. These 2 enzymes are thought to share a similar substrate spectrum,^22^ suggesting that CYP3A5 may also be involved in the metabolism of trazodone and the formation of mCPP. Both enzymes exhibit pronounced interindividual variability in enzyme activity. For CYP3A4, most of this variability is attributed to non-genetic factors. Although several single-nucleotide polymorphisms (SNPs) have been identified for *CYP3A4*, their clinical relevance often remains unclear or is substrate-specific. In contrast, the *CYP3A5* polymorphism rs776746 (*3 allele) has been well characterized.^23^ More than 90% of individuals of European descent are homozygous for this allele (*3/*3), which results in the absence of functional CYP3A5 enzyme expression in adults. Less than 10% of Caucasians carry at least one *1 allele, leading to CYP3A5 expression.^24,25^

In the case of *CYP2D6*, the impact of genetics on the enzyme’s activity is well established. Copy number variations and numerous SNPs are translating into CYP2D6 star alleles, which are applied to predict an individual’s metabolic phenotype. With this approach, individuals are classified into 4 categories: poor metabolizers (PM; no enzyme activity), intermediate metabolizers (IM; reduced enzyme activity), normal metabolizers (NM; normal enzyme activity), and ultra-rapid metabolizers (UM; increased enzyme activity).^26,27^

Nevertheless, a recently published review summarizing the results of 4 studies concluded that polymorphisms in *CYP2D6, CYP1A2, CYP3A4*, and *CYP3A5* do not affect the pharmacokinetics of trazodone or mCPP. However, a polymorphism (rs1045642) in the coding region of the *ATP-binding cassette sub-family B member 1 (ABCB1)* gene, coding for P-glycoprotein (P-gp), was found to influence the pharmacokinetics of trazodone plasma levels. The authors concluded that, due to the limited number of studies and their methodological limitations, further research is needed to assess the impact of pharmacogenetic markers on trazodone metabolism.^28^

To better understand the impact of genetic variants on trazodone and mCPP metabolism as well as on the tolerability of trazodone, we analyzed data from 2 ongoing pharmacogenetic studies. The primary goal of our research was to investigate a possible correlation between well-characterized pharmacogenetic markers—namely CYP3A5*3 and the CYP2D6 phenotype—and the occurrence of ADRs during trazodone treatment. Given the previously described associations between *ABCB1* polymorphisms and altered pharmacokinetics of trazodone, we also included *ABCB1* genotype data in our analysis.

## MATERIALS AND METHODS

### Study Design and Population

This is an exploratory observational study analyzing secondary data from 2 separate ongoing pharmacogenetic studies:

1. The “Pharmacist guided pre-emptive pharmacogenetic testing in antidepressant therapy” study (prePGx study, ClinicalTrials.gov Identifier: NCT04507555, EKNZ-ID: 2020-01535). This is a multicenter, open-label, randomized controlled trial investigating the service of pharmacist-guided pre-emptive pharmacogenetic testing on the response to antidepressants in adult inpatients diagnosed with a unipolar moderate or severe depressive episode.^29^
2. The “Pharmacogenetic Testing of Patients with Unwanted Adverse Drug Reactions or Therapy Failure” study (PGx Case Series study, ClinicalTrials.gov Identifier: NCT04154553, EKNZ-ID: 2019-01452). This is an observational study collecting health-related and pharmacogenetic data from patients who experienced therapy failure or ADR to previous medications.^30,31^

For this secondary data analysis, the inclusion criteria to use the patient data from the combined data pool were met if patients had taken trazodone before and/or at the time of study enrollment and/or during the study period. In addition, all included patients had to have signed a consent form for the further use of genetic data and biological material. No exclusion criteria were defined. The study was approved by the local ethics committee (EKNZ ID: 2024-00338, approved on March 8, 2024).

### Data Sources

In both source studies, patients received a commercially available pharmacogenetic panel test (Stratipharm by humatrix AG, Pfungstadt, Germany). The test includes more than 30 genes encoding for transport proteins, metabolizing enzymes, and drug targets (Supplemental Digital Content S1, http://links.lww.com/JCP/A998). Stratipharm provides an algorithm-based prediction of the phenotype based on the genotype. Patient medication history was documented at the time of study enrollment, including the names of the medications, dosages, and patient-reported information on ADRs. The medications and their dosages taken during the study were also recorded. In the prePGx study, ADRs were systematically collected and documented by physicians at weekly intervals for at least 4 weeks using Common Terminology Criteria for Adverse Events (CTCAE version 5.0).^32^ In the PGx Case Series study, patients were interviewed by a pharmacist about their current medications and their tolerability at 1 and 6 months in an open question format. Furthermore, serum samples were taken from patients in both studies. In the prePGx study, samples were collected at 2 and 4 weeks, and in the PGx Case Series study, they were collected at study enrollment. The procedures of these 2 studies are illustrated in Supplemental Digital Content S2, http://links.lww.com/JCP/A999.

### Data Extraction

The following patient-related data were extracted: sex, age at the time of enrollment in the prePGx study or PGx Case Series study, the daily dose of trazodone taken, and suspected ADRs attributed to trazodone. The time period of trazodone intake was often not recorded, as this information was mostly unavailable when trazodone had been taken before study inclusion. Adverse drug reactions were categorized according to MedDRA System Organ Classes (SOCs), as reported in the official product information of trazodone.

The genetic data included: *CYP2D6* diplotype (star allele) and the genotype-predicted phenotype (PM, IM, NM, and UM), and activity score (AS), *CYP3A5 rs776746* diplotype (star allele) and the genotype-predicted phenotype (PM (*3/*3), IM (*1/*3), NM (*1/*1)), and *ABCB1* diplotype (star allele) for rs1045642, rs1128503, rs2032582, and rs2032583. Detailed information on the SNPs analyzed and the CYP2D6 phenotype classification according to star allele nomenclature can be found in Supplemental Digital Content S3, http://links.lww.com/JCP/A1000.

In addition, co-medications (psychotropic and non-psychotropic medications) such as CYP2D6, CYP3A, and P-gp inhibitors and inducers taken concurrently with trazodone were recorded. These were identified using the *Carte Cytochromes (CYP) 2020* from the Department of Clinical Pharmacology and Toxicology, Geneva University Hospitals, Geneva, Switzerland, considering only potent inhibitors and inducers.^33^ To assess the phenoconverted CYP2D6 activity, the intake of CYP2D6 inhibitors was considered, and the phenoconverted CYP2D6 phenotype (pPM, pIM, pNM, and pUM) was calculated using the online available PROP™ Pharmacogenetics Calculator.^34^

### Serum Level Measurements

For a subgroup of patients, serum levels of trazodone and mCPP were analyzed according to routine procedures at the Institute of Clinical Chemistry, University Hospital Zurich, using a validated LC-MS/MS method (Exploris TLX system with APCI ionization, details available in Supplemental Digital Content S4, http://links.lww.com/JCP/B2). Serum samples were selected from patients with a daily trazodone dose ≥100 mg, if trazodone intake was confirmed at the time of collection and if trazodone serum level was assumed to be in steady state (more than 48 h of continuous intake). Given that trough levels could not be guaranteed, we investigated the dose-independent and time-independent ratio of mCPP serum levels to trazodone serum levels (mCPP:trazodone) for the different CYP2D6 phenotypes. We refrained from interpreting the mCPP:trazodone ratio in relation to *CYP3A5* genotype due to the small subgroup size and limited number of CYP3A5 IMs.

### Statistical Analysis

All the data were statistically analyzed using IBM SPSS software (version 29.0.2.0) and graphically presented using GraphPad Prism (version 10.2.0). We examined the differences in the occurrence of ADRs under trazodone for different genetic groups using the *χ²* test or Fisher exact tests (when appropriate). A sensitivity analysis was applied for all comparisons, categorizing patients whose tolerance or intolerance to trazodone was unclear (ADR unknown) into those with ADRs (ADR yes) or those without (ADR no). We interpreted the effect size Phi (φ) according to the following definition: φ<0.3 as a small effect, 0.3 to 0.5 as a moderate effect, and >0.5 as a large effect. A *P*-value <0.05 was considered as statistically significant. As our study setting was exploratory, we did not adjust for multiple testing.

We used logistic regression to investigate the effect of multiple variables on the occurrence of ADRs under trazodone therapy. As fixed effects in the model, we used the variables sex, trazodone dose, CYP2D6 phenotype after considering phenoconversion (pPM, pIM, and pNM), and CYP3A5 phenotype (PM, and IM and NM in one group together). Linearity was tested using the Box-Tidwell procedure. In addition, correlations between predictor variables were examined to ensure multicolinearity was not present. Outliers were checked, which were defined as studentized residuals >±3 SDs.

For serum level measurements, the Kruskal-Wallis test was used to analyze cross-group differences in the medians of serum levels for the different CYP2D6 phenotypes, both before and after phenoconversion. Dunn multiple comparison test with Bonferroni correction was performed to identify significant pairwise differences in the medians.

We tested Hardy-Weinberg equilibrium using Pearson *χ²* test for all genetic variants.

## RESULTS

### Demographics

**FIGURE 2:**
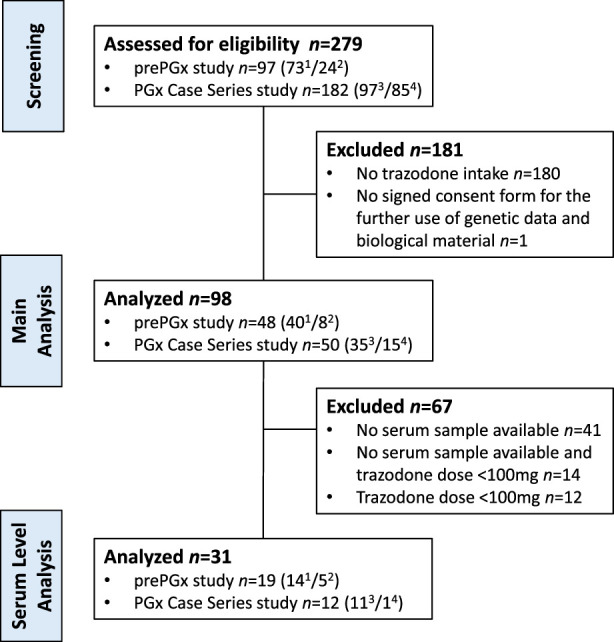
Study flowchart. ^1^ *n* patients from the study center Psychiatric Services Solothurn, Solothurn Hospitals, Solothurn, Switzerland. ^2^ *n* patients from the study center Wyss Private Clinic, Münchenbuchsee, Switzerland. ^3^ *n* patients from the study center Institute of Hospital Pharmacy, Solothurn Hospitals, Olten, Switzerland. ^4^ *n* patients from the study center Community Pharmacy, TopPharm Pharmacy am Spalebärg, Basel, Switzerland.

We screened a total of 97 patients in the prePGx study (from September 2020 to February 2024) and 182 patients in the PGx Case Series study (from October 2019 to February 2024) (Figure 2). Of these, 98 patients met the inclusion criteria and were included in this secondary analysis: 48 from the prePGx study (49% of those screened) and 50 from the PGx case series study (27.5%). These patients were predominantly female and middle-aged (Table 1). More than half of all patients reported ADRs from trazodone (55%), whereas reports on ADRs were unclear in 17% of participants. The most frequent ADRs belonged to the category of nervous system disorders (42%), followed by gastrointestinal disorders (27.5%) and psychiatric disorders (10.8%) (Table 2).

**TABLE 1:** Patient Demographics

	All Patients (N=98)	Subgroup With Serum Level Analysis (N=31)
Sex, n (%)		
Male	38 (39)	16 (52)
Female	60 (61)	15 (48)
Age, mean (±SD)	47.5 (±14.7)	45.2 (±13.5)
ADR, n (%)		
Yes	54 (55)	20 (65)
No	27 (28)	7 (23)
Unknown	17 (17)	4 (13)
Trazodone daily dose, n (%)		
<100 mg	26 (27)	0
100 mg	27 (28)	11 (35)
150-200 mg	22 (22)	12 (39)
>200 mg	11 (11)	8 (26)
Unknown	12 (12)	0
Trazodone intake, n (%)*		
Before study entry (history)	28 (29)	0
At study entry and during the study period	55 (56)	25 (81)
New during the study period	15 (15)	6 (19)
Source study, n (%)		
prePGx study	48 (49)	19 (61)
PGx case series study	50 (51)	12 (39)

* In relation to the source studies (prePGx and PGx case series).

ADR indicates adverse drug reaction.

**TABLE 2:** Adverse Drug Reactions From Trazodone According to MedDRA System Organ Classes

n tot (%)	102 (100)	Examples
Nervous system disorders	42 (41.2)	Tremor, headache, vertigo, hangover
Gastrointestinal disorders	28 (27.5)	Constipation, dry mouth, nausea
Psychiatric disorders	11 (10.8)	Restlessness, anxiety
Skin and subcutaneous tissue disorders	8 (7.8)	Hyperhidrosis, eczema, edema
Renal and urinary disorders	3 (2.9)	Urinary retention, urinary incontinence
Cardiac disorders	3 (2.9)	Hypertension, tachycardia
General disorders and administration site conditions	2 (2.0)	Feeling of weakness, malaise
Metabolism and nutrition disorders	2 (2.0)	Weight loss, weight gain
Vascular disorders	2 (2.0)	Orthostatic dysregulation
Hepatobiliary disorders	1 (1.0)	Increased liver values

All SNPs for *CYP3A5* and *ABCB1*, as well as the CYP2D6 genotype-predicted phenotype were in Hardy-Weinberg equilibrium, and their frequencies corresponded to those of a European reference population. Details on the distribution of the genetic markers in the studied population are shown in Table 3. Four different CYP2D6 inhibitors (duloxetine, fluoxetine, bupropion, and paroxetine) were recorded, which caused phenoconversion in 17 cases (Table 4). This resulted in an increased number of pPMs compared with genotype-predicted PMs, and correspondingly lower numbers of pNMs and pIMs compared with genotype-predicted NMs and IMs, respectively. No CYP3A inhibitors or CYP3A inducers were recorded. In 18 cases, a P-gp inhibitor (paroxetine, sertraline, fluoxetine, quetiapine, risperidone, and methadone) was taken. For details on all collected data, see Supplemental Digital Content S5, http://links.lww.com/JCP/B3.

**TABLE 3:** Distribution of Genotype-Predicted CYP2D6 Phenotype, CYP3A5 Phenotype, and ABCB1 Genotype

	All Patients (N=98)	Subgroup With Serum Level Analysis (N=31)
CYP2D6 genotype-predicted phenotype, n (%)		
PM	11 (11)	5 (16)
IM	41 (42)	17 (55)
NM	45 (46)	9 (29)
UM	1 (1)	0
CYP3A5 phenotype, n (%)		
PM (*3/*3)	87 (89)	27 (87)
IM (*1/*3)	10 (10)	4 (13)
NM (*1/*1)	1 (1)	0
ABCB1 rs1045642, n (%)		
C/C	22 (22)	6 (19)
C/T	51 (52)	16 (52)
T/T	25 (26)	9 (29)
ABCB1 rs1128503, n (%)		
C/C	36 (37)	13 (42)
C/T	41 (42)	10 (32)
T/T	21 (21)	8 (26)
ABCB1 rs2032582, n (%)		
G/G	33 (34)	12 (39)
G/T	42 (43)	11 (35)
T/T	19 (19)	7 (23)
G/A	3 (3)	1 (3)
A/T	1 (1)	0
ABCB1 rs2032583, n (%)		
C/C	0	0
C/T	22 (22)	5 (16)
T/T	76 (78)	26 (84)

IM indicates intermediate metabolizer; NM, normal metabolizer; PM, poor metabolizer; UM, ultra-rapid metabolizer.

**TABLE 4:** Distribution of Phenoconverted CYP2D6 Phenotype and CYP2D6 Phenoconversion in Relation to CYP2D6 Inhibitors

	All Patients (N=98)	Subgroup With Serum Level Analysis (N=31)
CYP2D6 phenoconverted phenotype, n (%)		
pPM	24 (24)	12 (39)
pIM	37 (38)	13 (42)
pNM	36 (37)	6 (19)
pUM	1 (1)	0
CYP2D6 phenoconversion, n (%)		
NM → pIM	4 (4)	1 (3)
*Duloxetine*	4 (4)	1 (3)
NM → pPM	5 (5)	2 (6)
*Fluoxetine*	1 (1)	1 (3)
*Bupropion*	3 (3)	1 (3)
*Paroxetine*	1 (1)	0
IM → pPM	8 (8)	5 (16)
*Fluoxetine*	1 (1)	0
*Bupropion*	5 (5)	4 (13)
*Paroxetine*	2 (2)	1 (3)

IM indicates intermediate metabolizer; NM, normal metabolizer; p, phenoconverted; PM, poor metabolizer; UM, ultra-rapid metabolizer.

### Association Between Pharmacogenetic Predictors and ADR

**FIGURE 3:**
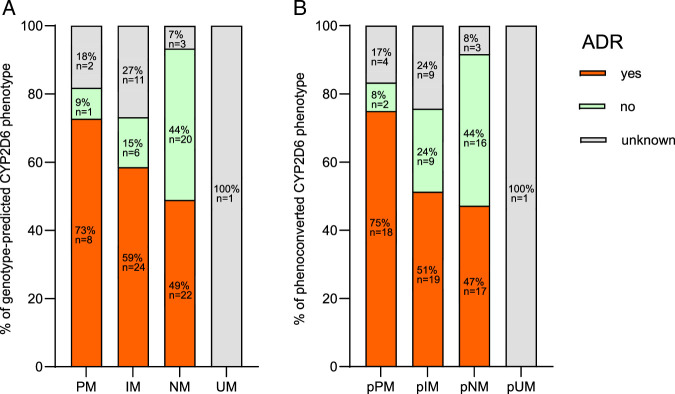
Percentage of adverse drug reaction by CYP2D6 phenotype. A, for genotype-predicted CYP2D6 phenotype. B, for phenoconverted CYP2D6 phenotype (after considering phenoconversion). ADR indicates adverse drug reaction; IM, intermediate metabolizer; NM, normal metabolizer; p, phenoconverted; PM, poor metabolizer; UM, ultra-rapid metabolizer. Color image is available online only at the journal’s website.

For the CYP2D6 genotype-predicted phenotype, there were signals that patients with reduced or missing CYP2D6 activity experienced relatively more ADRs compared with those with normal activity (Figure 3A). Comparing the relative frequency of ADRs for PMs and IMs together against NMs, a moderate effect was observed (φ=0.314, *P*=0.005). When comparing PMs and IMs separately against NMs, a small effect size was found (PMs vs. NMs: φ=0.283, *P*=0.064; IMs vs. NMs: φ=0.283, *P*=0.016).

When considering phenoconversion, the relative frequency of ADRs increased for pPMs and decreased for pIMs and pNMs (Figure 3B), resulting in a moderate effect when comparing the relative frequency of ADRs for pPMs with pNMs (φ=0.394, *P*=0.004). The comparison of pIMs to pNMs (φ=0.166, *P*=0.196) as well as the combined comparison of pPMs and pIMs to pNMs (φ=0.267, *P*=0.016) showed small effect sizes.

In the sensitivity analysis, when all patients for whom the tolerability or intolerance of trazodone was not clearly described were assigned to those with no ADRs, the observed trend was weakened or completely lost. No signals in the occurrence of ADRs were detected for any other group comparisons (CYP3A5 NM and IM vs. PM, or between the various *ABCB1* groups). For details on all performed statistical analyses, see Supplemental Digital Content S6, http://links.lww.com/JCP/B4.

For the logistic regression, after excluding patients with missing data for ADR (n=17) and/or trazodone dose (n=12), 72 patients were included in the analysis. No outliers were identified. After adjusting for the covariates sex, trazodone dose, and CYP3A5 phenotype, CYP2D6 pPMs were 9 times more likely to develop ADRs compared with pNMs (OR 8.96; 95% CI=1.67-48.08). No effect of sex, trazodone dose, and CYP3A5 phenotype on the occurrence of ADRs was observed after adjusting for all covariates, including CYP2D6 metabolizer status (after considering phenoconversion). All model coefficients and odds can be found in Supplemental Digital Content S7, http://links.lww.com/JCP/B5.

### Serum Level Measurements

For a subgroup of 31 patients, serum levels of trazodone and mCPP were measured, and the dose-independent ratio of mCPP to trazodone was calculated.

For the genotype-predicted CYP2D6 phenotypes, a trend toward higher median mCPP to trazodone ratios was observed in PMs compared with NMs (*P*=0.126), and in IMs compared with NMs (*P*=0.290), although these differences were not statistically significant (Table 5). The overall group difference before phenoconversion, assessed by the Kruskal-Wallis test, was not significant (*P*=0.1446).

**TABLE 5:** Median mCPP to Trazodone Ratio for Genotype-Predicted CYP2D6 Phenotype and Phenoconverted CYP2D6 Phenotype

mCPP:Trazodone, Median (95% CI)					
For Genotype-Predicted Phenotype			For Phenovconverted Phenotype		
PM, n=5	0.110 (0.070-0.370)	*P*=0.126	pPM, n=12	0.126 (0.074-0.292)	*P*=0.011
IM, n=17	0.080 (0.060-0.110)	*P*=0.290	pIM, n=13	0.075 (0.018-0.084)	*P*=0.874
NM, n=9	0.040 (0.030-0.090)		pNM, n=6	0.043 (0.034-0.087)	

*p*-values based on Dunn multiple comparison test with Bonferroni correction.

IM indicates intermediate metabolizer; mCPP, m-chlorophenylpiperazine; NM, normal metabolizer; p, phenoconverted; PM, poor metabolizer.

After accounting for phenoconversion, this trend became more pronounced. The mCPP to trazodone ratio was significantly higher in pPMs compared with pNMs (*P*=0.011), whereas the difference between pIMs and pNMs remained nonsignificant (*P*=0.874). The Kruskal-Wallis test indicated a significant overall group difference after phenoconversion (*P*=0.0072).

The median mCPP to trazodone ratios for the respective phenotypes all fall within the reference range of 0.04 to 0.22 provided in the literature, which includes all phenotypes.^35^ A detailed overview of serum level measurements is provided in Supplemental Digital Content S8, http://links.lww.com/JCP/B6.

**FIGURE 4:**
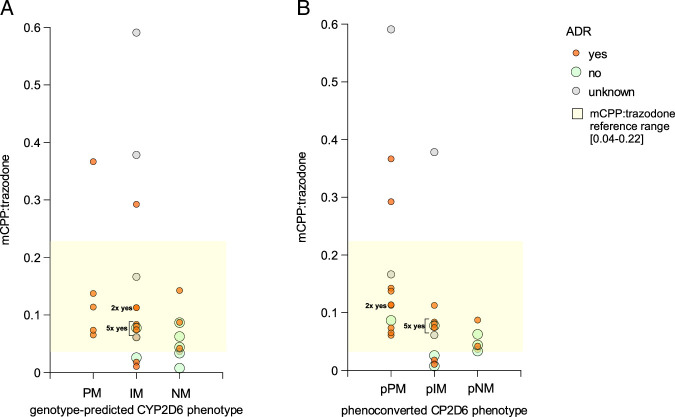
mCPP to trazodone ratio and adverse drug reaction according to CYP2D6 phenotype. A, For genotype-predicted CYP2D6 phenotype. B, for phenoconverted CYP2D6 phenotype (after considering phenoconversion). ADR indicates adverse drug reaction; IM, intermediate metabolize; mCPP, m-chlorophenylpiperazine; NM, normal metabolizer; p, phenoconverted; PM, poor metabolizer.

In addition, a trend was observed indicating that patients with a higher mCPP to trazodone ratio, particularly among PMs, experienced more side effects from trazodone (Figure 4). Here too, accounting for phenoconversion leads to a strengthening of this trend.

## DISCUSSION

In our exploratory analyses, we identified signals suggesting that CYP2D6 PMs and IMs exhibit a higher prevalence of ADRs from trazodone compared with NMs. This difference was further consolidated when considering phenoconversion by concurrent use of CYP2D6 inhibitors. After adjusting for sex, trazodone dose, and CYP3A5 phenotype, we observed an odds ratio of 8.96 for the occurrence of trazodone ADRs in CYP2D6 pPMs compared with pNMs. Furthermore, pPMs showed higher ratios of serum levels of mCPP to trazodone (mCPP:trazodone), which were also associated with a trend for a higher prevalence of ADRs. These results suggest that mCPP might be responsible for a relevant portion of trazodone-associated ADRs and that these could occur more frequently in patients with reduced CYP2D6 metabolism.

Our findings extend previous research that investigated the influence of the genotype-predicted CYP2D6 phenotype on mCPP plasma concentrations and found no significant correlations.^19,36^ Similarly, our own analyses did not reveal a statistically significant difference in the mCPP-to-trazodone ratio across CYP2D6 genotype-predicted phenotypes. However, a significant association became apparent after accounting for phenoconversion. Feuchtl et al^19^ genotyped 12 patients and identified 3 carriers of the CYP2D6*5 allele, which represents the loss of a gene copy. The authors did not further differentiate whether these were homozygous carriers (*5/*5, PMs) or heterozygous carriers (*1/*5, IMs). However, they did not observe any effect of the CYP2D6*5 allele on mCPP plasma concentrations. Mihara et al^36^ tested 54 Japanese patients for the non-function alleles *3, *4, *5, and the decreased function *10 allele, identifying 17 patients with *1/*1 (AS=2, NMs), 23 patients with *1/*10 (AS=1.25, NMs), 4 patients with *1/*5 (AS=1; IMs), 6 patients with *10/*10 (AS=0.5; IMs), 3 patients with *5/*10 (AS=0.25 IMs), and 1 patient with *4/*10 (AS=0.25; IM). They compared the mCPP plasma concentrations of the *1/*1 group (AS=2; NMs) with those carrying one mutated allele (AS=1.25, NMs and AS=1, IMs) and those carrying 2 mutated alleles (AS=0.5, IMs and 0.25; IMs). No differences between these groups were found. It should be noted, that the group classification did not correspond to the current definition of CYP2D6 phenotypes^37^ and no comparison was made with a CYP2D6 PM (AS=0) group. In this context, it is also important to mention that our own analysis of CYP2D6 genotype was limited to the SNPs included in the Stratipharm panel, and therefore did not cover all known variants relevant to CYP2D6 phenotyping. This represents a limitation of our study, as functionally important variants may have been missed.

A further study, conducted by Saiz-Rodriguez et al,^38^ investigated the influence of the CYP2D6 phenotype on the pharmacokinetics of trazodone and the occurrence of trazodone ADRs in 36 Caucasian patients. They found no difference in trazodone kinetics (maximum plasma concentration (C_max_); half-life (t_1/2_)) or exposure (trazodone area under the curve (AUC)), among different CYP2D6 phenotypes. However, CYP2D6 PMs showed an increased incidence of side effects like headache and paresthesia. As patients with more ADRs showed lower trazodone AUC and t_1/2_, the authors concluded that the ADRs might be due to a metabolite of trazodone. These observations align with our conclusion that mCPP could be responsible for some of the trazodone ADRs. Unfortunately, the pharmacokinetics of mCPP were not investigated in the study of Saiz-Rodriguez et al.^38^

Finally, a recently published case report describes trazodone-induced hepatotoxicity in a patient with CYP2D6 IM status. The authors concluded that the ADR could be attributed to the accumulation of mCPP.^39^

For mCPP, which is a substance responsible for a multitude of ADRs, previous documentation has led to warnings against the indiscriminate use of trazodone.^40^ Reported ADRs attributed to mCPP include physical symptoms such as nausea, sweating, hot and cold flushes, palpitations, tremor, dizziness, numbness, and chest pressure. Increased body temperature, elevated blood pressure, and elevated pulse rate have been measured in individual clinical studies. These physical symptoms are accompanied by an increase in serum ACTH, cortisol, and prolactin levels. In addition, mCPP can exacerbate anxiety and potentially induce panic attacks.^16^ Increased positive symptoms in schizophrenia patients have been described.^41^ Furthermore, a higher frequency of migraine attacks has been observed both in known migraine patients and in healthy individuals without a history of headaches.^42^ Similar ADRs were reported by some patients in our study, such as hyperhidrosis, tachycardia, tremor, and headaches as well as increased restlessness and anxiety. Hyperhidrosis and tachycardia were observed exclusively in patients with reduced CYP2D6 activity, which may lend support to the theory that mCPP is responsible for these ADRs (for details see Supplemental Digital Content S5, http://links.lww.com/JCP/B3). However, we were not able to definitively distinguish between ADRs caused by mCPP and those caused by trazodone itself. Unfortunately, we were not able to measure ACTH, cortisol, or prolactin levels in our study, which could have further substantiated our hypothesis that mCPP is contributing to these observed ADRs.

In addition to the fact that mCPP can cause numerous ADRs, pharmacokinetic studies have shown a large individual variability in mCPP absorption and elimination kinetics.^19,43^ This variability could be attributed to differences in CYP2D6 activity, which would further support our hypothesis that genetic variants in *CYP2D6* may influence serum levels of mCPP and, consequently, its potential to trigger ADRs.

Our results showed no association between the occurrence of trazodone ADRs and other genetic markers, such as genetic variants in *CYP3A5* and *ABCB1*. As CYP3A5 has a similar substrate spectrum as CYP3A4^22^ and may therefore contribute to the formation of mCPP, it seems plausible that genetic variants affecting *CYP3A5* could also play a role in trazodone tolerability. However, a significant portion of the metabolism is likely mediated by CYP3A4, which can also vary considerably in activity. As a result, the influence of *CYP3A5* genetics on trazodone metabolism may be diminished by the predominant activity of CYP3A4.^44^ To observe an association, it may be necessary to examine more patients, especially as CYP3A5 IMs and particularly NMs are very rare in the European population. Our study did not include an analysis of polymorphisms in the *CYP3A4* gene. Given the large number of possible SNPs and their often unclear clinical relevance, investigating *CYP3A4* genetics requires dedicated, well-controlled studies specifically designed to address this question. Findings from such studies could complement our results and contribute to a more comprehensive understanding of the genetic factors influencing trazodone metabolism.

For *ABCB1*, Saiz-Rodriguez et al^38,45^ found lower values for trazodone AUC, t_1/2_, and C_max_, along with a higher CL/F (total drug clearance adjusted for bioavailability) for individuals with the rs1045642 T/T genotype. In addition, T-allele carriers experienced more trazodone ADRs.^38^ In contrast, our results showed no difference in the occurrence of ADRs across different *ABCB1* SNPs, including rs1045642.

As mCPP is a potent 5-HT2C receptor agonist, it has been proposed that genetic variants in the *5-HT2C* gen could be responsible for interindividual differences in mCPP tolerability.^46^ In our study, we were not able to account for genetic variants in any 5-HT receptor to test this association. Future studies in this area should include polymorphisms in 5-HT receptor genes to better understand their potential role in influencing mCPP tolerability.

In addition to genetic predispositions, non-genetic factors can influence the pharmacokinetics of trazodone and mCPP, and consequently, their tolerability. These factors include trazodone dose, sex, age, BMI, and smoking status of the patients.^12^

Mihara and colleagues found that smokers exhibited significantly lower plasma concentrations of trazodone compared with non-smokers, whereas the difference in mCPP levels was not statistically significant. They hypothesized that CYP1A2 may be involved in trazodone metabolism, but found no association between CYP1A2 polymorphisms and plasma concentrations.^47^ Future studies should further investigate the potential role of CYP1A2, considering genetic variability.

Our results showed no effect of trazodone dose on the occurrence of ADRs, which is surprising as one would expect more ADRs with a higher dose. However, this could be due to the fact that in patients who experienced ADRs, trazodone dose was not further increased, thereby obscuring any potential association in our study.

A limitation of our study is the limited sample size and the imbalance in the patient population, with a higher proportion of female patients (60%). This is particularly important because sex-specific differences in trazodone pharmacokinetics^38^ and mCPP effects^48^ have been observed. As we only knew the age of the patients at the time of enrollment in the prePGx or PGx Case Series study, and this did not necessarily correspond to the age at which trazodone ADRs were experienced, we chose not to investigate age as a potential factor in the logistic regression analysis. BMI, smoking status, and comorbidities were not recorded in our study, which is a limitation. In addition, we did not distinguish the exact indication for trazodone use, whether it was used as an antidepressant or as a sleep medication. However, the high proportion of patients receiving low trazodone doses (over 50% received 100 mg or less) suggests that it was likely often used off-label for insomnia. This limits the generalizability of our findings to trazodone use as an antidepressant.

Another limitation of our study is that we analyzed data collected from 2 studies that were not designed to address our specific research question. In the PGx Case Series study, patients with ADRs were more frequently included, which may have led to a higher proportion of observed ADRs under trazodone treatment than what might be expected in the general population. Furthermore, the ADRs in these studies were self-reported by patients or observed by physicians, which can be unreliable, especially as patients were usually not on trazodone monotherapy. It is quite possible that they were taking other medications. Therefore, the experienced ADRs may not necessarily have been effects of trazodone or mCPP, but rather of the concomitant medications. For example, trazodone was often combined with other antidepressants, which can exhibit similar ADR profiles and are frequently metabolized by CYP2D6, potentially leading to accumulation in patients with reduced CYP2D6 activity. On the one hand, our observation that patients with a high mCPP: trazodone ratio experienced more side effects supports our theory that high mCPP levels would cause more ADRs. On the other hand, a high mCPP:trazodone ratio may simply reflect low CYP2D6 activity, which in turn could increase the risk of ADRs from other CYP2D6 substrates. Thus, our results need to be investigated and confirmed in further prospective studies.

## CONCLUSIONS

Our analyses indicate a higher risk of trazodone ADRs in patients with reduced CYP2D6 activity compared with normal metabolizers. As the trazodone metabolite mCPP is metabolized by CYP2D6 and the mCPP to trazodone ratio was increased in patients with CYP2D6 PM status, we hypothesize that mCPP could be responsible for some of the trazodone ADRs. Further large-scale clinical studies designed to evaluate this association are needed to confirm whether *CYP2D6* genotyping before prescribing trazodone could contribute to prevent ADRs in clinical practice.

## Supplementary Material
