# Supplementary material for: CYP2D6 Phenotype as a Predictor of Adverse Drug Reactions in Patients Treated With Trazodone: An Explorative Pharmacogenetic Study
Source: J Clin Psychopharmacol. 2026 Jan 7;46(2):179–88. doi: 10.1097/JCP.0000000000002123 (PMC12931868; doi:10.1097/JCP.0000000000002123)
Supplement: Supplementary file 1 [file jcp-46-179-s001.docx]

**CYP2D6 Phenotype as a Predictor of Adverse Drug Reactions in Patients Treated with Trazodone: An explorative Pharmacogenetic Study**

Supplement S1: Stratipharm® SNPs and annotations

| ***Gen*** | ***Chromosom / mitochondriale DNA*** | ***Annotation*** | ***Pos-Info*** | ***AS-Austausch*** | ***Basen*** |
| --- | --- | --- | --- | --- | --- |
| **ABCB1** | Chromosom 7q21.12 | **rs1045642** | NM_000927.4:c.3435T>C | I1145I | T>C |
| **ABCB1** | Chromosom 7q21.12 | **rs1128503** | NM_000927.4:c.1236T>C | G412G | T>C |
| **ABCB1** | Chromosom 7q21.12 | **rs2032582** | NM_000927.4:c.2677G>A | A893T | G>A |
| **ABCB1** | Chromosom 7q21.12 | **rs2032582** | NM_000927.4:c.2677G>T | A893S | G>T |
| **ABCB1** | Chromosom 7q21.12 | **rs2032583** | NM_000927.4:c.2685+49T>C | - | T>C |
| **ABCG2** | Chromosom 4q22-q23 | **rs2231142** | NM_004827.2:c.421C>A | Q141K | C>A |
| **ABCG2** | Chromosom 4q22-q23 | **rs13120400** | NM_004827.2:c.1194+928A>G | - | A>G |
| **ABCG2** | Chromosom 4q22-q23 | **rs17731538** | NC_000004.11:g.89055379G>A | - | G>A |
| **ADRB1** | Chromosom 10q24-q26 | **rs1801252** | NM_000684.2:c.145A>G | S49G | A>G |
| **ADRB1** | Chromosom 10q24-q26 | **rs1801253** | NM_000684.2:c.1165G>C | G389R | G>C |
| **ADRB2** | Chromosom 5q31-q32 | **rs1042713** | NT_029289.11:g.9369367G>A | G16R | G>A |
| **ADRB2** | Chromosom 5q31-q32 | **rs1042714** | NC_000005.9:g.148206473G>C | E27Q | G>C |
| **COMT** | Chromosom 22q11.21 | **rs4680** | NM_000754.3:c.472G>A | V158M | G>A |
| **COMT** | Chromosom 22q11.21 | **rs165599** | NM_000754.3:c.*522G>A | - | G>A |
| **COMT** | Chromosom 22q11.21 | **rs4646316** | NM_000754.3:c.615+310C>T | - | C>T |
| **COMT** | Chromosom 22q11.21 | **rs9332377** | NM_000754.3:c.616-367C>T | - | C>T |
| **COQ2** | Chromosom 4q21.23 | **rs4693075** | NC_000004.11:g.84192168G>C | - | G>C |
| **COQ2** | Chromosom 4q21.23 | **rs6535454** | NM_015697.7:c.894T>C | D298D | T>C |
| **CYP1A2** | Chromosom 15q24.1 | **rs2069514** | NC_000015.9:g.75038220G>A | - | G>A |
| **CYP1A2** | Chromosom 15q24.1 | **rs762551** | NC_000015.9:g.75041917C>A | - | C>A |
| **CYP2B6** | Chromosom 19q13.2 | **rs8192709** | NM_000767.4:c.64C>T | R22C | C>T |
| **CYP2B6** | Chromosom 19q13.2 | **rs28399499** | NM_000767.4:c.983T>C | I328T | T>C |
| **CYP2B6** | Chromosom 19q13.2 | **rs3745274** | NM_000767.4:c.516G>T | Q172H | G>T |
| **CYP2C8** | Chromosom 10q24.1 | **rs10509681** | NM_000770.3:c.1196A>G | K399R | A>G |
| **CYP2C8** | Chromosom 10q24.1 | **rs11572080** | NM_000770.3:c.416G>A | R139K | G>A |
| **CYP2C8** | Chromosom 10q24.1 | **rs1934951** | NG_007972.1:g.35707G>A | - | G>A |
| **CYP2C9** | Chromosom 10q24.1 | **rs1799853** | NM_000771.3:c.430C>T | R144C | C>T |
| **CYP2C9** | Chromosom 10q24.1 | **rs1057910** | NM_000771.3:c.1075A>C | I359L | A>C |
| **CYP2C9** | Chromosom 10q24.1 | **rs9332131** | NM_000771.3:c.817delA | K273X | delA |
| **CYP2C9** | Chromosom 10q24.1 | **rs7900194** | NM_000771.3:c.449G>A | R150H | G>A |
| **CYP2C9** | Chromosom 10q24.1 | **rs28371685** | NM_000771.3:c.1003C>T | R335W | C>T |
| **CYP2C19** | Chromosom 10q24 | **rs4244285** | NM_000769.1:c.681G>A | - | G>A |
| **CYP2C19** | Chromosom 10q24 | **rs4986893** | NM_000769.1:c.636G>A | W212X | G>A |
| **CYP2C19** | Chromosom 10q24 | **rs12248560** | NG_008384.1:g.4195C>T | - | C>T |
| **CYP2C19** | Chromosom 10q24 | **rs28399504** | NM_000769.1:c.1A>G | M1V | A>G |
| **CYP2D6** | Chromosom 22q13.1 | **-** | copy number variation | - | CNV |
| **CYP2D6** | Chromosom 22q13.1 | **rs35742686** | NM_000106.4:c.775delA | - | delA |
| **CYP2D6** | Chromosom 22q13.1 | **rs3892097** | NM_000106.4:c.506-1G>A | - | G>A |
| **CYP2D6** | Chromosom 22q13.1 | **rs5030655** | NM_000106.4:c.454delT | - | delT |
| **CYP2D6** | Chromosom 22q13.1 | **rs5030867** | NM_000106.4:c.971A>C | H324P | A>C |
| **CYP2D6** | Chromosom 22q13.1 | **rs5030865** | NM_000106.4:c.505G>T | G169X | G>T |
| **CYP2D6** | Chromosom 22q13.1 | **rs5030865** | NM_000106.4:c.505G>A | G169R | G>A |
| **CYP2D6** | Chromosom 22q13.1 | **rs5030656** | NM_000106.5:c.841_843delAAG | K281del | delAAG |
| **CYP2D6** | Chromosom 22q13.1 | **rs1065852** | NM_000106.4:c.100C>T | P34S | C>T |
| **CYP2D6** | Chromosom 22q13.1 | **rs201377835** | NM_000106.5:c.181-1G>C | - | G>C |
| **CYP2D6** | Chromosom 22q13.1 | **rs28371706** | NM_000106.4:c.320C>T | T107I | C>T |
| **CYP2D6** | Chromosom 22q13.1 | **rs59421388** | NM_000106.4:c.1012G>A | V338M | G>A |
| **CYP2D6** | Chromosom 22q13.1 | **rs28371725** | NM_000106.4:c.985+39G>A | - | G>A |
| **CYP3A4** | Chromosom 7q21.1 | **rs2740574** | NG_000004.3:g.135607G>A | - | G>A |
| **CYP3A4** | Chromosom 7q21.1 | **rs2242480** | NM_017460.5:c.1026+12G>A | - | G>A |
| **CYP3A5** | Chromosom 7q21.1 | **rs776746** | NM_000777.3:c.219-237G>A | - | G>A |
| **DPYD** | Chromosom 1p22 | **rs3918290** | NM_000110.3:c.1905+1G>A | - | G>A |
| **DPYD** | Chromosom 1p22 | **rs72549303** | NM_000110.3:c.1898delC | - | delC |
| **DPYD** | Chromosom 1p22 | **rs72549309** | NM_000110.3:c.298delTinsTCAT | - | delTinsTCAT |
| **DPYD** | Chromosom 1p22 | **rs55886062** | NM_000110.3:c.1679T>G | I560S | T>G |
| **DPYD** | Chromosom 1p22 | **rs67376798** | NM_000110.3:c.2846A>T | D949V | A>T |
| **DPYD** | Chromosom 1p22 | **rs2297595** | NM_000110.3:c.496A>G | M166V | A>G |
| **GNB3** | Chromosom 12p13 | **rs5443** | NM_002075.2:c.825C>T | S275S | C>T |
| **GSTP1** | Chromosom 11q13.2 | **rs1695** | NM_000852.3:c.313A>G | I105V | A>G |
| **HLA-A** | Chromosom 6p21.3 | **rs1061235** | NM_002116.7:c.*66A>T | - | A>T |
| **HLA-A** | Chromosom 6p21.3 | **rs1633021** | NC_000006.12:g.29779092T>C | - | T>C |
| **HLA-B** | Chromosom 6p21.3 | **rs3909184** | NM_005803.2:c.724-507C>G | - | C>G |
| **HLA-B** | Chromosom 6p21.3 | **rs2395029** | NM_006674.3:c.*568T>G | - | T>G |
| **HLA-B** | Chromosom 6p21.3 | **rs2844682** | NC_000006.11:g.30946148G>A | - | G>A |
| **HMGCR** | Chromosom 5q13.3-q14 | **rs17238540** | NM_000859.2:c.2457+117T>G | - | T>G |
| **HMGCR** | Chromosom 5q13.3-q14 | **rs17244841** | NM_000859.2:c.451-174A>T | - | A>T |
| **HTR2A** | Chromosom 13q14-q21 | **rs6311** | NC_000013.10:g.47471478C>T | - | C>T |
| **HTR2A** | Chromosom 13q14-q21 | **rs6313** | NM_000621.3:c.102C>T | S34S | C>T |
| **HTR2A** | Chromosom 13q14-q21 | **rs7997012** | NM_000621.3:c.614-2211T>C | - | T>C |
| **HTR2A** | Chromosom 13q14-q21 | **rs9316233** | NC_000013.10:g.47433355C>G | - | C>G |
| **HTR2A** | Chromosom 13q14-q21 | **rs6314** | NC_000013.10:g.47409034G>A | H368Y | G>A |
| **IFNL3** | Chromosom 19q13.13 | **rs8099917** | NC_000019.9:g.39743165T>G | - | T>G |
| **IFNL3** | Chromosom 19q13.13 | **rs12979860** | NC_000019.9:g.39738787C>T | - | C>T |
| **ITPA** | Chromosom 20p | **rs1127354** | NM_181493.1:c.43C>A | P32T | C>A |
| **MT-RNR1** | mitchondriale DNA | **rs267606617** | NC_012920.1:m.1555A>G | - | A>G |
| **NAT2** | Chromosom 8p22 | **rs1801280** | NM_000015.2:c.341T>C | I114T | T>C |
| **NAT2** | Chromosom 8p22 | **rs1799930** | NM_000015.2:c.590G>A | R197Q | G>A |
| **NAT2** | Chromosom 8p22 | **rs1799931** | NM_000015.2:c.857G>A | G286E | G>A |
| **OPRM1** | Chromosom 6q24-q25 | **rs1799971** | NM_000914.3:c.118A>G | N40D | A>G |
| **SLC19A1** | Chromosom 21q22.3 | **rs1051266** | NM_194255.1:c.80A>G | H27R | A>G |
| **SLCO1B1** | Chromosom 12p12 | **rs4149056** | NM_006446.4:c.521T>C | V174A | T>C |
| **SLCO1B1** | Chromosom 12p12 | **rs11045819** | NM_006446.4:c.463C>A | P155T | C>A |
| **SLCO1B1** | Chromosom 12p12 | **rs2306283** | NM_006446.4:c.388A>G | N130D | A>G |
| **SLCO1B1** | Chromosom 12p12 | **rs4149015** | NG_011745.1:g.4195G>A | - | G>A |
| **TPMT** | Chromosom 6p22.3 | **rs1800462** | NM_000367.2:c.238G>C | A80P | G>C |
| **TPMT** | Chromosom 6p22.3 | **rs1800460** | NM_000367.2:c.460G>A | A154T | G>A |
| **TPMT** | Chromosom 6p22.3 | **rs1142345** | NM_000367.2:c.719A>G | Y240C | A>G |
| **TPMT** | Chromosom 6p22.3 | **rs1800584** | NM_000367.2:c.626-1G>A | - | G>A |
| **TPMT** | Chromosom 6p22.3 | **rs12201199** | NM_000367.2:c.419+94T>A | - | T>A |
| **VKORC1** | Chromosom 16p11.2 | **rs9923231** | NC_000016.9:g.31107689C>T | - | C>T |
| **VKORC1** | Chromosom 16p11.2 | **rs7294** | NM_024006.4:c.*134G>A | - | G>A |
| **VKORC1** | Chromosom 16p11.2 | **rs17708472** | NM_024006.4:c.173+525C>T | - | C>T |
| **VKORC1** | Chromosom 16p11.2 | **rs2359612** | NM_024006.4:c.283+837T>C | - | T>C |
| **VKORC1** | Chromosom 16p11.2 | **rs8050894** | NM_024006.4:c.283+124G>C | - | G>C |
| **VKORC1** | Chromosom 16p11.2 | **rs9934438** | NM_024006.4:c.174-136C>T | - | C>T |
