# Supplementary material for: CYP2D6 Phenotype as a Predictor of Adverse Drug Reactions in Patients Treated With Trazodone: An Explorative Pharmacogenetic Study
Source: J Clin Psychopharmacol. 2026 Jan 7;46(2):179–88. doi: 10.1097/JCP.0000000000002123 (PMC12931868; doi:10.1097/JCP.0000000000002123)
Supplement: Supplementary file 2 [file jcp-46-179-s002.docx]

**CYP2D6 Phenotype as a Predictor of Adverse Drug Reactions in Patients Treated with Trazodone: An explorative Pharmacogenetic Study**

**Supplement S2: Study design of the prePGx-Study and PGx Case Series Study**


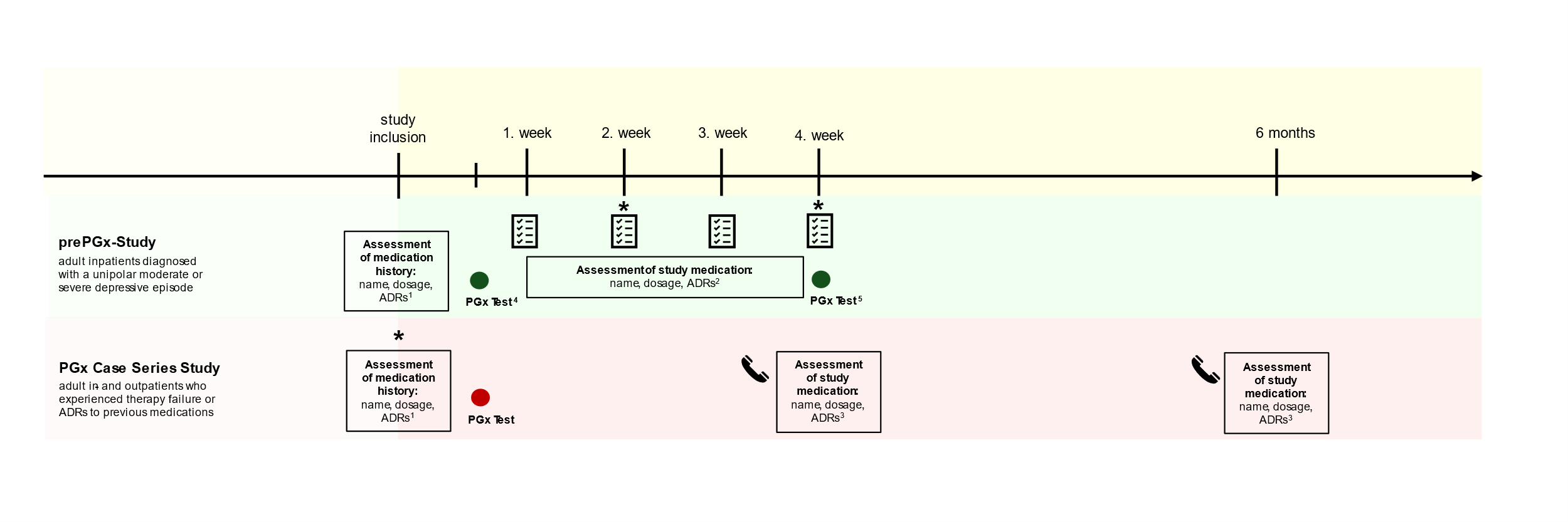


**Study design of the prePGx-Study and PGx Case Series Study. PGx: Pharmacogenetic, ADRs: Adverse drug reactions. * Collection of** serum samples, ^1^ ADRs were assessed by study personal in an open question format, ^2^ ADRs were assessed by physicians using Common Terminology Criteria for Adverse Events (CTCAE version 5.0), ^3^ ADRs were assessed by pharmacists in an open question format during a phone call, ^4^ patients in the PGx-guided arm of the prePGx-Study received PGx test results at study baseline, ^5^ patients in the control arm of the prePGx-Study received PGx test results after four weeks
