# Supplementary material for: CYP2D6 Phenotype as a Predictor of Adverse Drug Reactions in Patients Treated With Trazodone: An Explorative Pharmacogenetic Study
Source: J Clin Psychopharmacol. 2026 Jan 7;46(2):179–88. doi: 10.1097/JCP.0000000000002123 (PMC12931868; doi:10.1097/JCP.0000000000002123)
Supplement: Supplementary file 3 [file jcp-46-179-s003.docx]

**CYP2D6 Phenotype as a Predictor of Adverse Drug Reactions in Patients Treated with Trazodone: An explorative Pharmacogenetic Study**

**Supplement S3:**

Studied single nucleotide polymorphisms in ABCB1, CYP2D6 and CYP3A5

| ***Gene*** | ***Chromosome / mitochondrial DNA*** | ***Annotation*** | ***Pos-Information*** | ***Star Alleles*** | ***AS-Exchange*** | ***Base*** |
| --- | --- | --- | --- | --- | --- | --- |
| **ABCB1** | Chromosome 7q21.12 | **rs1045642** | NM_000927.4:c.3435T>C | - | I1145I | T>C |
| **ABCB1** | Chromosome 7q21.12 | **rs1128503** | NM_000927.4:c.1236T>C | - | G412G | T>C |
| **ABCB1** | Chromosome 7q21.12 | **rs2032582** | NM_000927.4:c.2677G>A | - | A893T | G>A |
| **ABCB1** | Chromosome 7q21.12 | **rs2032582** | NM_000927.4:c.2677G>T | - | A893S | G>T |
| **ABCB1** | Chromosome 7q21.12 | **rs2032583** | NM_000927.4:c.2685+49T>C | - | - | T>C |
| **CYP2D6** | Chromosome 22q13.1 | **-** | copy number variation | - | - | CNV |
| **CYP2D6** | Chromosome 22q13.1 | **rs35742686** | NM_000106.4:c.775delA | *3 | - | delA |
| **CYP2D6** | Chromosome 22q13.1 | **rs3892097** | NM_000106.4:c.506-1G>A | *4 | - | G>A |
| **CYP2D6** | Chromosome 22q13.1 | **rs5030655** | NM_000106.4:c.454delT | *6 | - | delT |
| **CYP2D6** | Chromosome 22q13.1 | **rs5030867** | NM_000106.4:c.971A>C | *7 | H324P | A>C |
| **CYP2D6** | Chromosome 22q13.1 | **rs5030865** | NM_000106.4:c.505G>T | *8 | G169X | G>T |
| **CYP2D6** | Chromosome 22q13.1 | **rs5030865** | NM_000106.4:c.505G>A | *14, *114 | G169R | G>A |
| **CYP2D6** | Chromosome 22q13.1 | **rs5030656** | NM_000106.5:c.841_843delAAG | *9 | K281del | delAAG |
| **CYP2D6** | Chromosome 22q13.1 | **rs1065852** | NM_000106.4:c.100C>T | *4, *10, *114 | P34S | C>T |
| **CYP2D6** | Chromosome 22q13.1 | **rs201377835** | NM_000106.5:c.181-1G>C | *11 | - | G>C |
| **CYP2D6** | Chromosome 22q13.1 | **rs28371706** | NM_000106.4:c.320C>T | *17 | T107I | C>T |
| **CYP2D6** | Chromosome 22q13.1 | **rs59421388** | NM_000106.4:c.1012G>A | *29 | V338M | G>A |
| **CYP2D6** | Chromosome 22q13.1 | **rs28371725** | NM_000106.4:c.985+39G>A | *41 | - | G>A |
| **CYP3A5** | Chromosome 7q21.1 | **rs776746** | NM_000777.3:c.219-237G>A | *3 | - | G>A |

CYP2D6 phenotype classification according to star allele nomenclature and activity score

**CYP2D6 PMs**

| **Diplotyp** | **Activity Score** |
| --- | --- |
| *3/*3 | 0 |
| *3/*4 | 0 |
| *3/*5 | 0 |
| *3/*6 | 0 |
| *4/*4 | 0 |
| *4/*5 | 0 |
| *4/*6 | 0 |
| *5/*5 | 0 |
| *5/*6 | 0 |
| *5/*7 | 0 |
| *5/Ex9Conv | 0 |
| *3&*4&*10/*4 | 0 |
| *3/*3&*41 | 0 |
| *4&*10&*11/*11 | 0 |
| *3/*4x2 | 0 |
| *3x2/*4 | 0 |
| *4/Ex9Conv | 0 |
| *4/*4x2 | 0 |
| *4/*4 (+1 Ex9Conv) | 0 |
| *4/*4 (+2 Ex9Conv) | 0 |
| *4/*6 (+1 In1Conv) | 0 |
| *3/Ex9Conv | 0 |
| *4/*4x3 | 0 |
| *4/*4&*9&*10 | 0 |
| *4/*7 | 0 |
| *4/In1Conv | 0 |

**CYP2D6 IMs**

| **Diplotyp** | **Activity Score** |
| --- | --- |
| *1/*11 | 1 |
| *1/*3 | 1 |
| *1/*3 (+1 In1Conv) | 1 |
| *1/*3+*4 | 1 |
| *1/*3x2 | 1 |
| *1/*4 | 1 |
| *1/*4 (+1 Ex9Conv) | 1 |
| *1/*4 (+1 In1Conv) | 1 |
| *1/*4 (+2 Ex9Conv) | 1 |
| *1/*4 (+2 In1Conv) | 1 |
| *1/*4x2 | 1 |
| *1/*4x3 | 1 |
| *1/*4x4 | 1 |
| *1/*5 | 1 |
| *1/*6 | 1 |
| *1/*6x3 | 1 |
| *1/*7 | 1 |
| *1/*8 | 1 |
| *1/Ex9Conv | 1 |
| *1/Ex9Conv (+1 In1Conv) | 1 |
| *10&*41/Ex9Conv | 0.25 |
| *10/*10 | 0.5 |
| *10/*10 (+1 Ex9Conv) | 0.5 |
| *10/*10 (+2 Ex9Conv) | 0.5 |
| *10/*29 | 0.75 |
| *10/*41 | 0.75 |
| *10/Ex9Conv | 0.25 |
| *10/Ex9Conv (+1 In1Conv) | 0.25 |
| *11/*41 | 0.5 |
| *17/*17 | 1 |
| *17/*29 | 1 |
| *17/*41 | 1 |
| *29/*29 | 1 |
| *3/*10 | 0.25 |
| *3/*4+*10 | 0.25 |
| *3/*41 | 0.5 |
| *3/*9 | 0.5 |
| *4&*10&*17/*17 | 0.5 |
| *4&*10&*41/*10 | 0.25 |
| *4/*10 | 0.25 |
| *4/*10 (+1 Ex9Conv) | 0.25 |
| *4/*10 (+2 Ex9Conv) | 0.25 |
| *4/*10&*41 | 0.25 |
| *4/*10+*41 | 0.75 |
| *4/*10x2 | 0.5 |
| *4/*10x3 | 0.75 |
| *4/*10x4 | 1 |
| *4/*17 | 0.5 |
| *4/*41 | 0.5 |
| *4/*41x2 | 1 |
| *4/*9 | 0.5 |
| *4/*9 (+2 Ex9Conv) | 0.5 |
| *4/*9+*10 | 0.75 |
| *4/*9x2 | 1 |
| *41/*41 | 1 |
| *41/Ex9Conv | 0.5 |
| *41/In1Conv | 0.5 |
| *4x2/*10 | 0.25 |
| *4x2/*10x2 | 0.5 |
| *4x2/*10x3 | 0.75 |
| *4x2/*41 | 0.5 |
| *4x2/*9 | 0.5 |
| *4x3/*10 | 0.25 |
| *4x3/*10x2 | 0.5 |
| *4x4/*10 | 0.25 |
| *5/*10 | 0.25 |
| *5/*29 | 0.5 |
| *5/*41 | 0.5 |
| *5/*9 | 0.5 |
| *6/*10 | 0.25 |
| *6/*41 | 0.5 |
| *6/*9 | 0.5 |
| *7/*41 | 0.5 |
| *9/*10 | 0.75 |
| *9/*10 (+2 Ex9Conv) | 0.75 |
| *9/*41 | 1 |
| *9/*41 (+1 In1Conv) | 1 |
| *9/*9 | 1 |
| *9/*9 (+2 Ex9Conv) | 1 |
| *9/Ex9Conv (+2 In1Conv) | 0.5 |

**CYP2D6 NMs**

| **Diplotyp** | **Activity Score** |
| --- | --- |
| *1/*1 | 2 |
| *1/*1 (+1 Ex9Conv) | 2 |
| *1/*1 (+1 In1Conv) | 2 |
| *1/*1 (+2 Ex9Conv) | 2 |
| *1/*1 (+5 In1Conv) | 2 |
| *1/*10 | 1.25 |
| *1/*10 (+1 Ex9Conv) | 1.25 |
| *1/*10 (+2 Ex9Conv) | 1.25 |
| *1/*10x2 | 1.5 |
| *1/*10x3 | 1.75 |
| *1/*14 | 1.5 |
| *1/*17 | 1.5 |
| *1/*17x2 | 2 |
| *1/*29 | 1.5 |
| *1/*4+*10 | 1.25 |
| *1/*4+*10x2 | 1.5 |
| *1/*4+*10x3 | 1.75 |
| *1/*4+*41 | 1.5 |
| *1/*4+*9 | 1.5 |
| *1/*41 | 1.5 |
| *1/*41 (+1 In1Conv) | 1.5 |
| *1/*41x2 | 2 |
| *1/*4x2+*10 | 1.25 |
| *1/*4x2+*10x2 | 1.5 |
| *1/*4x3+*10 | 1.25 |
| *1/*9 | 1.5 |
| *1/*9 (+1 In1Conv) | 1.5 |
| *1/*9 (+2 Ex9Conv) | 1.5 |
| *1/*9x2 | 2 |
| *10/*10x4 | 1.25 |
| *1x2/*10 | 2.25 |
| *1x2/*3 | 2 |
| *1x2/*4 | 2 |
| *1x2/*4+*10 | 2.25 |
| *1x2/*4x2 | 2 |
| *1x2/*4x2+*10 | 2.25 |
| *1x2/*4x3 | 2 |
| *1x2/*6x2 | 2 |

**CYP2D6 UMs**

| **Diplotyp** | **Activity Score** |
| --- | --- |
| *1/*17x3 | 2.5 |
| *1/*1x2 | 3 |
| *1/*1x2 (+1 Ex9Conv) | 3 |
| *1/*1x2 (+1 In1Conv) | 3 |
| *1/*1x3 | 4 |
| *1/*1x4 | 5 |
| *1/*1x5 | 6 |
| *1/*41x3 | 2.5 |
| *1/*41x5 | 3.5 |
| *1/*9x3 | 2.5 |
| *1x2/*10x2 | 2.5 |
| *1x2/*17 | 2.5 |
| *1x2/*17x2 | 3 |
| *1x2/*4+*10x2 | 2.5 |
| *1x2/*41 | 2.5 |
| *1x2/*41x2 | 3 |
| *1x2/*41x4 | 4 |
| *1x2/*9 | 2.5 |
| *1x2/*9x2 | 3 |
| *1x3/*10 | 3.25 |
| *1x3/*17 | 3.5 |
| *1x3/*4 | 3 |
| *1x3/*4+*10 | 3.25 |
| *1x3/*41 | 3.5 |
| *1x3/*41x3 | 4.5 |
| *1x3/*4x2 | 3 |
| *1x3/*6 | 3 |
| *1x3/*9 | 3.5 |
| *1x4/*4 | 4 |
| *1x4/*41x2 | 5 |
| *1x5/*41 | 5.5 |
|  |  |
