# Supplementary material for: CYP2D6 Phenotype as a Predictor of Adverse Drug Reactions in Patients Treated With Trazodone: An Explorative Pharmacogenetic Study
Source: J Clin Psychopharmacol. 2026 Jan 7;46(2):179–88. doi: 10.1097/JCP.0000000000002123 (PMC12931868; doi:10.1097/JCP.0000000000002123)
Supplement: Supplementary file 4 [file jcp-46-179-s004.docx]

**CYP2D6 Phenotype as a Predictor of Adverse Drug Reactions in Patients Treated with Trazodone: An explorative Pharmacogenetic Study**

**Supplement S4: LC-MS/MS Method for Trazodone and mCPP Quantification**

Serum concentrations of trazodone and meta-chlorophenylpiperazine (mCPP) were measured using an LC-MS/MS method with atmospheric pressure chemical ionization (APCI) on an **Exploris TLX system**.

**Chromatographic Conditions**

- **Extraction column:** Cyclone 50 × 0.5 mm
- **Analytical column:** Uptisphere 5 µm ODB 125 × 2 mm
- **Injection volume:** 30 µL
- **Run time:** ~10 minutes

**Mobile Phases**

- **Mobile Phase A (Sirolimus/Everolimus A):** 1.54 g ammonium acetate dissolved in 2 L water, plus 2 mL concentrated formic acid; mix thoroughly.
- **Mobile Phase B (Sirolimus/Everolimus B):**
  - Prepare stock solution: 7.7 g ammonium acetate in 1 L methanol, shake well.
  - Final mix: 50 mL of stock solution + 200 mL methanol + 250 mL acetonitrile + 1 mL concentrated formic acid; shake well.
- **Mobile Phase C ("Magic Mix"):** 1500 mL acetone + 1500 mL 2-propanol + 1500 mL acetonitrile; shake well.

**Retention Times**

- **Trazodone:** ~5.69 minutes
- **mCPP:** ~5.42 minutes

**Quantifier Ions (m/z)**

- **Trazodone:** 372.15856
- **mCPP:** 197.08400

**Calibration and Quantification Limits**

- **LLOQ (Lower Limit of Quantification):**
  - Trazodone: 0.135 µmol/L (plus standard 0 at 0 µmol/L)
  - mCPP: 0.051 µmol/L (plus standard 0 at 0 µmol/L)
- **ULOQ (Upper Limit of Quantification):**
  - Trazodone: 6.73 µmol/L
  - mCPP: 2.55 µmol/L

**Precision**

- **Trazodone:** Coefficient of variation (CV) 6% at 5.8 µmol/L
- **mCPP:** CV 6.6% at 0.15 µmol/L
