# Supplementary material for: CYP2D6 Phenotype as a Predictor of Adverse Drug Reactions in Patients Treated With Trazodone: An Explorative Pharmacogenetic Study
Source: J Clin Psychopharmacol. 2026 Jan 7;46(2):179–88. doi: 10.1097/JCP.0000000000002123 (PMC12931868; doi:10.1097/JCP.0000000000002123)
Supplement: Supplementary file 6 [file jcp-46-179-s006.docx]

**CYP2D6 Phenotype as a Predictor of Adverse Drug Reactions in Patients Treated with Trazodone: An explorative Pharmacogenetic Study**

**Supplement S6:** χ² test or Fisher’s exact test (when at least one cell has an expected frequency <5) for different group comparison

| n tot = 98 | specificity analysis | | | sensitivity analysis with unknown ADR = ADR yes | | | sensitivity analysis with unknown ADR = ADR no | | |
| --- | --- | --- | --- | --- | --- | --- | --- | --- | --- |
| group comparison | **% ADR yes within the group** *(n)* | **% ADR no within the group** *(n)* | **p-value** (Phi coefficients (φ) or Cramér's V (V)) | **% ADR yes within the group** *(n)* | **% ADR no within the group** *(n)* | **p-value** (Phi coefficients (φ) or Cramér's V (V)) | **% ADR yes within the group** *(n)* | **% ADR no within the group** *(n)* | **p-value** (Phi coefficients (φ) or Cramér's V (V)) |
| CYP2D6 PM vs. CYP2D6 NM | **73** (8) vs **49** (22) | **9** (1) vs **44** (20) | **0.064^f^** (0.283) | **91** (10) vs **56** (25) | **9** (1) vs **44** (20) | **0.039^f^** (0.290) | **73** (8) vs **49** (22) | **27** (3) vs **51** (23) | **0.155** (0.190) |
| CYP2D6 IM vs. CYP2D6 NM | **59** (24) vs **49** (22) | **15** (6) vs **44** (20) | **0.016** (0.283) | **85** (35) vs **56** (25) | **15** (6) vs **44** (20) | **0.003** (0.324) | **59** (24) vs **49** (22) | **41** (17) vs **51** (23) | **0.370** (0.097) |
| CYP2D6 PM&IM vs. CYP2D6 NM | **62** (32) vs **49** (22) | **13** (7) vs **44** (20) | **0.005** (0.314) | **87** (45) vs **56** (25) | **13** (7) vs **44** (20) | **<0.001*** (0.345) | **62** (32) vs **49** (22) | **38** (20) vs **51** (23) | **0.211** (0.127) |
| CYP2D6 pPM vs. CYP2D6 pNM | **75** (18) vs **47** (17) | **8** (2) vs **44** (16) | **0.004** (0.394) | **82** (22) vs **56** (20) | **8** (2) vs **44** (16) | **0.003** (0.386) | **75** (18) vs **47** (17) | **25** (6) vs **53** (19) | **0.033** (0.276) |
| CYP2D6 pIM vs. CYP2D6 pNM | **51** (19) vs **47** (17) | **24** (9) vs **44** (16) | **0.196** (0.166) | **76** (28) vs **56** (20) | **24** (9) vs **44** (16) | **0.070** (0.212) | **51** (19) vs **47** (17) | **49** (18) vs **53** (19) | **0.724** (0.041) |
| CYP2D6 pPM&pIM vs. CYP2D6 pNM | **61** (37) vs **47** (17) | **18** (11) vs **44** (16) | **0.016** (0.267) | **82** (50) vs **56** (20) | **18** (11) vs **44** (16) | **0.005** (0.285) | **61** (37) vs **47** (17) | **39** (24) vs **53** (19) | **0.198** (0.131) |
| CYP3A5 NM&IM vs. CYP3A5 PM | **55** (6) vs **55** (48) | **45** (5) vs **25** (22) | **0.493^f^** (0.102) | **55** (6) vs **75** (65) | **45** (5) vs **25** (22) | **0.169^f^** (0.142) | **55** (6) vs **55** (48) | **45** (5) vs **45** (39) | **1.000^f^** (0.004) |
| ABCB1 rs1045642 C/C vs. C/T vs. T/T | **55** (12) vs **55** (28) vs **56** (14) | **23** (5) vs **29** (15) vs **28** (7) | **0.921** (0.045) | **77** (17) vs **71** (36) vs **72** (18) | **23** (5) vs **29** (15) vs **28** (7) | **0.841** (0.060) | **55** (12) vs **55** (28) vs **56** (14) | **45** (10) vs **29** (23) vs **44** (11) | **0.994** (0.011) |
| ABCB1 rs1128503 C/C vs. C/T vs. T/T | **61** (22) vs **54** (22) vs **48** (10) | **25** (8) vs **29** (12) vs **29** (6) | **0.802** (0.074) | **75** (27) vs **71** (29) vs **71** (15) | **25** (8) vs **29** (12) vs **29** (6) | **0.910** (0.044) | **61** (22) vs **54** (22) vs **48** (10) | **39** (14) vs **46** (19) vs **52** (11) | **0.596** (0.103) |
| ABCB1 rs2032582 G/G vs. G/T vs. T/T vs. G/A vs. A/T | **55** (18) vs **55** (23) vs **53** (10) vs **100** (3) vs **0** (0) | **30** (10) vs **29** (12) vs **26** (5) vs **0** (0) vs **0** (0) | **0.818^f^** (0.140) | **70** (23) vs **71** (30) vs **74** (14) vs **100** (3) vs **100** (1) | **30** (10) vs **29** (12) vs **26** (5) vs **0** (0) vs **0** (0) | **0.934^f^** (0.131) | **55** (18) vs **55** (23) vs **53** (10) vs **100** (3) vs **0** (0) | **45** (15) vs **45** (19) vs **47** (9) vs **0** (0) vs **100** (1) | **0.524^f^** (0.195) |
| ABCB1 rs2032583 C/T vs. T/T | **55** (12) vs **55** (42) | **27** (6) vs **28** (21) | **1.000** (0.000) | **73** (16) vs **72** (55) | **27** (6) vs **28** (21) | **0.974** (0.003) | **55** (12) vs **55** (42) | **45** (10) vs **45** (34) | **0.952** (0.006) |

^f^ At least one cell has an expected frequency <5.
