# Supplementary material for: CYP2D6 Phenotype as a Predictor of Adverse Drug Reactions in Patients Treated With Trazodone: An Explorative Pharmacogenetic Study
Source: J Clin Psychopharmacol. 2026 Jan 7;46(2):179–88. doi: 10.1097/JCP.0000000000002123 (PMC12931868; doi:10.1097/JCP.0000000000002123)
Supplement: Supplementary file 7 [file jcp-46-179-s007.docx]

**CYP2D6 Phenotype as a Predictor of Adverse Drug Reactions in Patients Treated with Trazodone: An explorative Pharmacogenetic Study**

**Supplement S7: Logistic Regression Analyses**

CYP2D6 after considering phenoconversion (PMa = pPM; IMa = pIM, EMa = pEM)
