# Supplementary material for: CYP2D6 Phenotype as a Predictor of Adverse Drug Reactions in Patients Treated With Trazodone: An Explorative Pharmacogenetic Study
Source: J Clin Psychopharmacol. 2026 Jan 7;46(2):179–88. doi: 10.1097/JCP.0000000000002123 (PMC12931868; doi:10.1097/JCP.0000000000002123)
Supplement: Supplementary file 8 [file jcp-46-179-s008.docx]

**CYP2D6 Phenotype as a Predictor of Adverse Drug Reactions in Patients Treated with Trazodone: An explorative Pharmacogenetic Study**

**Supplement S8**

Trazodone (TZD) Dose [mg], Trazodone serum concentration [umol/l], mCPP serum concentration [umol/l], and ratio mcPP:TZD

CYP2D6 phenotype before considering phenoconversion:

CYP2D6 phenotype after considering phenoconversion:

CYP3A5 phenotype

ABCB1 rs1045642

ABCB1 rs1128503

ABCB1 rs2032582

ABCB1 rs2032583
